# Traditional East Asian herbal medicines for the treatment of poststroke constipation: A protocol for systematic review and meta analysis

**DOI:** 10.1097/MD.0000000000025503

**Published:** 2021-04-16

**Authors:** Chul Jin, Bo-Hyoung Jang, Jin Pyeong Jeon, Ye-Seul Lee, Seung-Bo Yang, Seungwon Kwon

**Affiliations:** a Department of Cardiology and Neurology; b Department of Preventive Medicine, College of Korean Medicine, Kyung Hee University, Seoul; c Department of Neurosurgery, Chuncheon Sacred Heart Hospital, Hallym University Medical Center, Hallym University College of Medicine, Chuncheon; d Jaseng Spine and Joint Research Institute, Jaseng Medical Foundation, Seoul; e Department of Korean Internal Medicine, College of Korean Medicine, Gachon University, Seongnam, Republic of Korea.

**Keywords:** herbal medicine, post-stroke constipation, stroke, systematic review

## Abstract

**Background:**

Post-stroke constipation is a major complication of stroke and increases the incidence of poor neurological outcomes and infectious complications and, therefore, warrants active and prompt treatment. In East Asian countries, several types of herbal medicines have been used for the treatment of post-stroke constipation because they are considered safer than existing pharmacotherapies. However, no systematic review has investigated the efficacy and safety of traditional East Asian herbal medicine in the treatment of post-stroke constipation. With this systematic review and meta-analysis, we aimed to evaluate the efficacy and safety of traditional East Asian herbal medicines for the treatment of post-stroke constipation.

**Methods and analysis:**

Eight electronic databases will be searched for relevant studies published from inception to April 2021. Only randomized controlled trials (RCTs) that assess the efficacy and safety of traditional East Asian herbal medicines for the treatment of post-stroke constipation will be included in this study. The methodological qualities, including the risk of bias, will be evaluated using the Cochrane risk of bias assessment tool. After screening the studies, a meta-analysis of the RCTs will be performed, if possible.

**Results:**

This study is expected to generate high-quality evidence of the efficacy and safety of herbal medicines to treat post-stroke constipation.

**Conclusion:**

Our systematic review will provide evidence to determine whether herbal medicines can be effective interventions for patients with post-stroke constipation.

**Ethics and dissemination:**

Ethical approval is not required, as this study was based on a review of published research. This review will be published in a peer-reviewed journal and disseminated electronically and in print.

**Trial registration number:**

Research registry reviewregistry1117

## 1 Introduction

Post-stroke constipation is a major complication after stroke and has been reported to occur in 22.9–79% of patients with stroke.^[1,2]^ Discomfort due to constipation causes distress in both patients and their caregivers, and can negatively affect the patient's quality of life.^[3,4]^ Furthermore, post-stroke constipation increases the length of hospital stay, confers poor rehabilitation outcome, increases the recurrence of stroke, and can cause death in patients with stroke;^[1,5]^ furthermore, it has been reported to increase the incidence of infectious complications, such as pneumonia and urinary tract infections.^[1]^ Therefore, active and prompt treatment of post-stroke constipation is essential.

Currently, pharmacotherapies, such as laxatives (osmotic and stimulant), anticholinesterases, enterokinetic medications, secretagogues, and serotonin 5-HT4 receptor agonists, have been mainly used to treat post-stroke constipation.^[6,7]^ However, these medications are known to cause adverse effects, including electrolyte imbalance, nausea, headache, diarrhea, abdominal pain, anaphylaxis, and carcinogenesis.^[6,8]^ Therefore, there is a shortage of effective strategies for the treatment of constipation in patients with stroke, mostly elderly patients. In addition, long-term use of conventional pharmacotherapies can cause dependence and permanent changes in the bowel habits of patients with stroke.^[4,8]^

These limitations of existing therapies warrant the need to develop safer and more effective treatments for post-stroke constipation. Traditional medicine, which mainly uses herbs, acupuncture, and moxibustion, is still widely used in Northeast Asian regions, such as Korea, China, Japan, and Taiwan,^[9–11]^ and several related studies on the effects of traditional medicine to treat functional constipation or post-stroke constipation have steadily emerged. Dahuang (Rhei Radix et Rhizoma) is the most commonly used herb for treating constipation.^[12]^ A prospective, double-blind, double-dummy, randomized controlled trial^[13]^ suggested that MaZiRenWan, which contains Dahuang, could be effective to treat functional constipation. An open-label study reported that another herbal prescription, Daikenchuto, which does not contain Dahuang, significantly improve the constipation score (constipation scoring system [CSS]) in patients with post-stroke constipation.^[14,15]^ In order for decision makers to easily utilize these existing evidence in the clinical setting, a systematic review is needed to identify, evaluate, and summarize related studies.^[16]^ However, to date, no systematic review has been conducted to evaluate the efficacy and safety of traditional East Asian herbal medicine to treat post-stroke constipation.

Therefore, the aims of this study are as follows:

(1) To assess whether traditional East Asian herbal medicine therapies for the treatment of post-stroke constipation are more effective and safer than conventional Western medicine therapies or placebo.
(2) To assess whether adjunct traditional East Asian herbal medicine therapies in combination with conventional Western medicine therapies is more effective and safer than conventional Western medicine therapies alone for the treatment of post-stroke constipation.

## 2 Methods

### 2.1 Study registration

The protocol of the present study adhered to the Preferred Reporting Items for Systematic Reviews and Meta-Analysis Protocol (PRISMA) guidelines and checklist^[17]^ and has been registered with the Research Registry 2021 under number review registry1117.

### 2.2 Eligible criteria for study selection

#### 2.2.1 Types of studies

Only randomized controlled trials (RCTs) investigating the efficacy and safety of traditional East Asian herbal medicines for the treatment of post-stroke constipation will be included in this study, without any publication or language restrictions. Quasi-randomized controlled trials (such as those allocating participants by alternate days of the week or date of birth), non-RCTs, case reports, case series, uncontrolled trials, and laboratory studies will be excluded. Studies that fail to provide detailed results will be excluded. Cross-over trials will also be excluded because of the potential for a carry-over effect.

#### 2.2.2 Types of participants

Eligible participants will be defined as adult patients (over 18 years of age) having constipation after a life-first or recurrent stroke. Post-stroke constipation should be diagnosed according to at least one of the current diagnostic criteria or diagnostic criteria at the time of the study. Patients with a history of constipation before the diagnosis of stroke will be excluded. There will be no restrictions based on sex, ethnicity, symptom severity, disease duration, and clinical setting. However, patients with subdural hemorrhage or subarachnoid hemorrhage will be excluded.

#### 2.2.3 Types of interventions

We will include studies using traditional East Asian herbal medicines alone or adjunct traditional East Asian herbal medicines in combination with conventional Western medicine therapies as experimental interventions. In the present study, only oral administration forms of traditional East Asian herbal medicines will be included, with no limitations on the dosage, frequency, duration of treatment, and formulation (decoctions, extracts, tablets, capsules, and powders). Therefore, intravenous or acupuncture point injections of herbal medicines will be excluded.

The control interventions will include placebo, placebo + conventional Western medicine therapies, or conventional Western medicine therapies alone. We will exclude studies comparing other traditional East Asian medicine therapies, such as those using different types of traditional East Asian herbal medicines, acupuncture, or moxibustion. Studies comparing the effect of traditional East Asian herbal medicines with other traditional East Asian medicine therapies, such as acupuncture treatment or moxibustion, will also be excluded.

#### 2.2.4 Types of outcome measures

For the primary outcome, we will assess the frequency of spontaneous defecation, defined as the mean number of spontaneous defecations per week. For secondary outcomes, we will include the CCS and gas volume score (calculated by Koide's method ^[18]^), the frequency of use of rescue medications (laxatives or rectal evacuants), mean transit time, total effective rate for post-stroke constipation, and other parameters evaluating neurologic deficits, such as the National Institute of Health Stroke Scale score, modified Rankin Scale (mRS) score, modified Barthel Index (mBI), and quality of life (QoL). We will also investigate the number and severity of adverse events.

### 2.3 Search strategies for the identification of studies

#### 2.3.1 Electronic searches

The following electronic databases will be searched from inception to April 2021: MEDLINE (via PubMed), the Cochrane Central Register of Controlled Trials (CENTRAL), Excerpta Medica dataBASE (EMBASE), Scopus, Citation Information by Nii (CiNii), China National Knowledge Infrastructure Database (CNKI), Oriental Medicine Advanced Searching Integrated System (OASIS), and National Digital Science Library (NDSL). The specific search strategies (for example, PubMed) are listed in Table 1.

**Table 1.** Search strategy for PubMed.

#1	Search (“stroke” [mesh] OR “cerebral apoplexy” [tiab] OR “cerebrovascular disease” [tiab] OR “cerebrovascular accident” [tiab] OR “cerebral infarction” [tiab] OR “cerebral hemorrhage” [tiab] OR “ischemic stroke” [tiab] OR “hemorrhagic stroke” [tiab] OR “intracerebral hemorrhage” [tiab])
#2	Search (“constipation” [mesh] OR “constipation” [tiab] OR “constipated” [tiab] OR “constipating” [tiab] OR “constipations” [tiab])
#3	Search (“herbal med∗” [tiab] OR “herbal com∗” [tiab] OR “herb∗” [tiab])
#4	Search (“traditional kor∗” [tiab] OR “korean med∗” [tiab])
#5	Search “traditional chin∗” [tiab]
#6	Search (“kanpo” [tiab] OR “kampo” [tiab])
#7	Search “decoction ” [tiab]
#8	#3 or #4 or #5 or #6 or #7
#9	Search (“randomise∗” [tiab] OR “randomize∗” [tiab])
#10	#1 and #2 and #8 and #9
#11	Search (“animals [Mesh] NOT “humans [Mesh])
#12	#10 NOT #11

We will make relative modifications in accordance with the requirements, and an equivalent translation of the search terms will be adopted to ensure that similar search terms are used in all databases. If additional information is needed from the identified studies, we will contact the corresponding authors.

#### 2.3.2 Search for other resources

A manual search will also be performed to search the reference lists of the relevant articles. Clinical trial registries (ClinicalTrials.gov, Clinical Research Information Service [CRIS]), conference presentations, and expert contacts will also be searched.

### 2.4 Data collection and analysis

#### 2.4.1 Study selection

**Figure 1:**
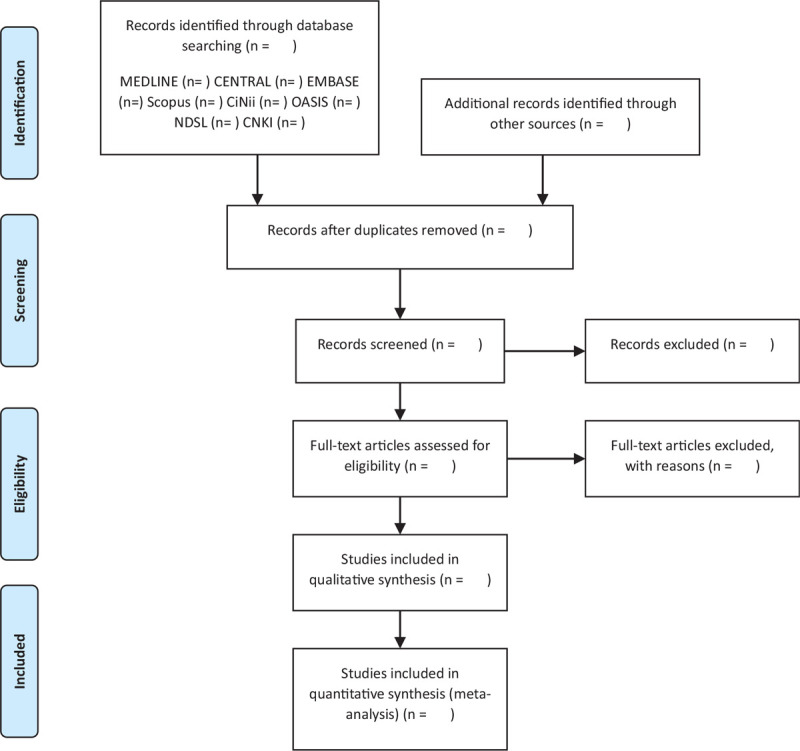
A PRISMA flow diagram of literature screening and selection processes.

Two reviewers (SK and CJ) trained in the process and purpose of study selection will independently review the titles, abstracts, and manuscripts of the studies and screen them for eligibility for inclusion in the analysis. After removing duplicates, the full texts will be reviewed. All studies, identified by both electronic and manual searches, will be uploaded to EndNote X9 (Clarivate Analytics), and the reasons for excluding studies will be recorded and shown in a PRISMA flowchart, as shown in Figure 1. All disagreements will be resolved by consulting an independent reviewer (BHJ).

#### 2.4.2 Data extraction and management

One review writer (CJ) will independently extract the data and fill out the standard data extraction form, which includes study information—the first author, publication year, language, sample size, characteristics of participants (e.g., age, sex, and types of stroke), details of randomization, blinding, interventions (names of herbal medicines used, type of formula, number and dosage of administration), comparison (types of comparison [e.g., placebo, no additional treatment, number and dosage of administration]), treatment period, outcome measures, primary outcome, secondary outcome, and statistical method used. Another independent review writer (SW) will confirm the contents of the extraction. Disagreements, if any, will be resolved by consulting another review writer (BHJ).

#### 2.4.3 Assessment of bias risk and quality of included studies

Two reviewers (SK and CJ) will assess the risk of bias (RoB) based on the Cochrane Collaboration tool,^[19]^ which includes references to random sequence generation (selection bias), allocation concealment (selection bias), blinding of participants and personnel (performance bias), blinding of outcome assessment data (detection bias), incomplete outcome data (attribution bias), selective reporting (reporting bias), and other biases. The assessment results will be presented as follows: low, unclear, and high RoB.

#### 2.4.4 Measurement of treatment effect

For continuous data, the pooled results will be presented as the mean difference (MD) or standardized MD with 95% confidence intervals (CIs). For dichotomous data, the pooled results will be presented as a risk ratio (RR) with 95% CIs.

#### 2.4.5 Managing missing data

If there are missing, insufficient, or unclear data, we will contact the corresponding author and gather relevant information. If the information cannot be obtained, only the remaining available information, which will be discussed, will be analyzed.

#### 2.4.6 Assessment of heterogeneity

We will perform the I^2^ test to evaluate statistical heterogeneity. Statistical heterogeneity will be considered if I^2^ is greater than 50%.

#### 2.4.7 Data synthesis

The Review Manager program (V.5.4 Copenhagen: The Nordic Cochrane Center. The Cochrane Collaboration, 2014) will be used for statistical analysis. If I^2^ is ≤ 50%, the fixed-effect model will be used to evaluate the outcome data. Otherwise, a random-effects model will be used. The studies will be synthesized according to the type of intervention and/or control as follows:

1. Traditional East Asian herbal medicines vs. conventional Western medicine therapies
2. Traditional East Asian herbal medicines vs. placebo
3. Traditional East Asian herbal medicines + conventional Western medicine therapies vs. placebo + conventional Western medicine therapies
4. Traditional East Asian herbal medicines + conventional Western medicine therapies vs. conventional Western medicine therapies alone.

The heterogeneity levels will be assessed in the included literature, and if enough studies are available to investigate the causes of heterogeneity and its criteria, the groups indicated below (Subgroup analysis section) will be assessed. If more than 10 studies are included in the meta-analysis, we will estimate publication bias using Egger's test and depict the results visually with a funnel plot. We will use the Grading of Recommendations Assessment, Development and Evaluation (GRADE) pro software from Cochrane Systematic Reviews to create a Summary of Findings table.

#### 2.4.8 Subgroup analysis

If sufficient studies are available to investigate the cause of heterogeneity and its criteria, the following will be assessed: the types of stroke (e.g., ischemic or hemorrhagic), stroke duration (e.g., acute or chronic), the name of the herbal medicines used, and the formula of the herbal medicine (such as granules or decoctions).

#### 2.4.9 Sensitivity analysis

We will perform a sensitivity analysis to verify the robustness of the results. This will be done by assessing the impact of sample size, high RoB, missing data, and selected models. Following the analyses, if the quality of the studies is judged to be low, these studies will be removed to ensure the robustness of the results.

#### 2.4.10 Ethics and dissemination

A formal ethical approval was not required for this protocol. We will collect and analyze data based on published studies, and because no patients are directly or specifically assessed in this study, individual privacy will not be a concern. The results of this review will be disseminated to peer-reviewed journals or presented at a relevant conference.

## 3 Discussion

Post-stroke constipation can negatively impact the prognosis of patients with stroke.^[1,3–5]^ It not only leads to poor QoL in patients with stroke but also increases the prevalence of complications, such as pneumonia and urinary tract infection.^[1,3–5]^ However, currently used pharmacological treatments have a one-off effect and adverse effects, such as electrolyte imbalance and anaphylaxis, which could be fatal to patients with stroke;^[6,8]^ thus, the necessity for the development of new treatments continues to increase.

Clinical trials have reported that MaZiRenWan (which contains Dahuang)^[13]^ and Daikenchuto (without Dahuang)^[14,15]^ could be effective in treating functional constipation. Both herbal prescriptions are herbal combinations with a long history of use and listed in the “Synopsis of Prescriptions of the Golden Chamber, ” published during the Han Dynasty in ancient China; these formulations have been used to improve constipation. The pharmacological mechanisms underlying the clinical effects of these two prescriptions have also been reported. In a previous study,^[20]^ the focused network pharmacology approach was used to analyze the mechanism of action of MaZiRenWan on constipation; the study found that representative compounds of MaZiRenWan, such as amygdalin, albiflorin, emodin, honokiol, and naringin, could induce spontaneous contractions of colonic smooth muscle. Furthermore, several previous studies have suggested that Zanthoxylum fruit, one of the components of Daikenchuto, could improve delayed propulsion in the small intestine and distal colon,^[21]^ while maltose, another component, induces endogenous cholecystokinin secretion,^[21]^ both of which reportedly helps to improve constipation. Thus, traditional East Asian herbal medicines are likely to become newer alternatives to existing Western medicines to improve post-stroke constipation.

The current review will be conducted to assess the efficacy and safety of using herbal medicine to treat post-stroke constipation and establish novel management strategies that is expected to reduce the burden on patients and their caregivers.

## Author contributions

**Conceptualization:** Seungwon Kwon.

**Data curation:** Bo-Hyoung Jang, Jin Pyeong Jeon, Ye-Seul Lee, Seung-Bo Yang, Seungwon Kwon.

**Formal analysis:** Bo-Hyoung Jang, Jin Pyeong Jeon, Ye-Seul Lee, Seungwon Kwon.

**Funding acquisition:** Seungwon Kwon.

**Project administration:** Seungwon Kwon.

**Writing – original draft:** Chul Jin, Seungwon Kwon.

**Writing – review & editing:** Chul Jin, Bo-Hyoung Jang, Jin Pyeong Jeon, Ye-Seul Lee, Seung-Bo Yang, Seungwon Kwon.

## Correction

The grant number appeared incorrectly as HB20C0147 and has been corrected to HF20C0147.
